# Arrhythmic mitral valve prolapse: valve geometry and traction force quantification by echocardiography

**DOI:** 10.1093/europace/euae224

**Published:** 2024-08-27

**Authors:** Sofía Capdeville, Raúl González Sánchez, Álvaro Velasco, Rafael Salguero-Bodes, Fernando Arribas Ynsaurriaga, Jorge Solís

**Affiliations:** Department of Cardiology, Hospital Universitario 12 de Octubre, Avenida de Córdoba S/N, Madrid CP: 28041, Spain; Instituto de Investigación Sanitaria Hospital 12 de Octubre (imas12), Madrid, Spain; Grupo de Bioingeniería y Telemedicina, Escuela técnica Superior de Ingenieros de Telecomunicación, Madrid, Spain; Department of Cardiology, Hospital Universitario 12 de Octubre, Avenida de Córdoba S/N, Madrid CP: 28041, Spain; Department of Cardiology, Hospital Universitario 12 de Octubre, Avenida de Córdoba S/N, Madrid CP: 28041, Spain; Instituto de Investigación Sanitaria Hospital 12 de Octubre (imas12), Madrid, Spain; Centro de Investigación Biomédica en Red de Enfermedades Cardiovasculares (CIBERCV), Instituto de Salud Carlos III, Avda. Monforte de Lemos 5-7, 28029 Madrid, Spain; Department of Cardiology, Hospital Universitario 12 de Octubre, Avenida de Córdoba S/N, Madrid CP: 28041, Spain; Instituto de Investigación Sanitaria Hospital 12 de Octubre (imas12), Madrid, Spain; Centro de Investigación Biomédica en Red de Enfermedades Cardiovasculares (CIBERCV), Instituto de Salud Carlos III, Avda. Monforte de Lemos 5-7, 28029 Madrid, Spain; Department of Cardiology, Hospital Universitario 12 de Octubre, Avenida de Córdoba S/N, Madrid CP: 28041, Spain; Instituto de Investigación Sanitaria Hospital 12 de Octubre (imas12), Madrid, Spain; Centro de Investigación Biomédica en Red de Enfermedades Cardiovasculares (CIBERCV), Instituto de Salud Carlos III, Avda. Monforte de Lemos 5-7, 28029 Madrid, Spain

**Keywords:** Mitral valve prolapse, Arrhythmic mitral valve prolapse, Mitral valve geometry, Traction forces

Identifying the subgroup of patients with mitral valve prolapse (MVP) and high incidence of ventricular arrhythmias (VA) represents a challenge. The arrhythmic MVP (AMVP) has been defined as the presence of MVP, combined with frequent and/or complex VA in the absence of any other arrhythmic substrate, regardless of the presence of mitral regurgitation (MR).^1–4^ There have been efforts to identify echocardiographic characteristics of AMVP, being bileaflet prolapse and mitral annular disjunction (MAD) the most studied ones.^5,6^ It has been previously postulated that leaflet displacement exerts increased tension on papillary muscles (PMs) causing excessive traction,^7^ which may be linked to the reduced regional strain and myocardial fibrosis at the PMs and basal inferior-lateral left ventricular (LV) wall, that is seen in this population.^8–10^

We hypothesize that mitral valve apparatus geometry and subsequent excessive traction forces on the PMs constitute the underlying pathophysiological pathway for the development of VA. We believe we are able to identify and quantify this mechanism by transthoracic echocardiography (TTE). Therefore, by identifying patients with disruptive traction forces, we could define echocardiographic patterns associated with VA.

This is a retrospective cohort study. Forty-two patients (*n* = 42) were selected for a proof-of-concept analysis. Mitral valve prolapse was defined as systolic displacement of one or both mitral leaflets ≥ 2 mm above the plane of the mitral annulus (MA) in the parasternal long-axis view and AMVP as MVP combined with frequent or complex VA [≥5% total premature ventricular contraction (PVC) burden, non-sustained ventricular tachycardia (NSVT), ventricular tachycardia, or ventricular fibrillation].^1^ Valve geometry and traction parameters were measured using 2D TTE. Traction forces were quantified as the change in distance between PM and MA from early to peak systole (*Figure 1*).^7^

**Figure 1:**
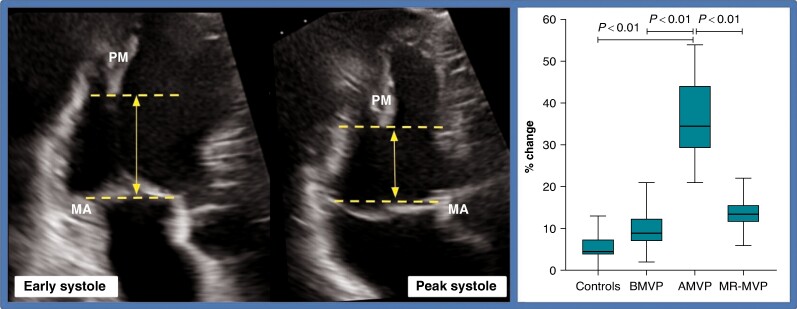
Left panel: traction was measured in a three-chamber apical view as the distance from the tip of the PM to a line at the level of the mitral annular hinge points at 2 moments: (i) at full closure of the mitral valve (early systole) and (ii) in mid-late systole at the time of maximal superior leaflet displacement (peak systole). The change in this distance between the two moments was calculated and represents traction. Right panel: PM-MA distance change (traction) in the four groups. AMVP, arrhythmic MVP; BMVP, benign MVP; MA, mitral annulus; MR-MVP, MVP with severe mitral regurgitation; MVP, mitral valve prolapse; PM, papillary muscle.

The patients were divided into four groups: a control group (controls, *n* = 10), a group with benign MVP with no significant MR (BMVP, *n* = 14), an AMVP group with no significant MR (AMVP, *n* = 8), and a group with MVP and severe MR (MR-MVP, *n* = 10).

Clinical characteristics and echo measurements are presented in *Table 1*. The groups were comparable in terms of age, gender and LV dimension, function, and global longitudinal strain. Palpitations were more common in AMVP, while dyspnoea was the predominant symptom in MR-MVP. The AMVP group also had a higher likelihood of pathological electrocardiogram (ECG), with PVCs and negative T waves being the most common findings. The AMVP patients exhibited the highest burden of VA on 24 h Holter monitoring, predominantly NSVT. As expected, MR-MVP patients had the largest left atrium volume. In terms of mitral valve geometry, the AMVP group had the largest prolapsing area and height, with primarily bileaflet involvement, and MAD was significantly more frequent in this group as well (six out of the eight patients had MAD).

**Table 1:** Basal patients’ characteristics and echocardiographic measurements

	Total	Controls	BMVP^a^	AMVP^b^	MR-MVP	*P* value	Controls vs. BMVP	Controls vs. AMVP	Controls vs. MR-MVP	BMVP vs. AMVP	BMVP vs. MR-MVP	AMVP vs. MR-MVP
Basal characteristics												
Patients, *n*	42 (100%)	10 (23.8%)	14 (33.3%)	8 (19.1%)	10 (23.8%)							
Age, years	47.7 (16.9)	42.3 (13.1)	48.1 (17.8)	45.5 (14.4)	54.4 (20.6)	0.45	0.39	0.59	0.20	0.76	0.38	0.31
Gender (men), *n*	16 (38.1%)	4 (40%)	5 (35.7%)	3 (37.5%)	4 (40%)	1.00	0.83	0.91	1.00	0.93	0.83	0.91
BMI, kg/m^2^	23.7 (3.8)	25.1 (1.9)	21.9 (3.6)	22.2 (3.2)	26.3 (4.3)	*0*.*010*	*0*.*026*	0.062	0.88	0.73	*0*.*019*	0.051
Symptoms, *n*						0.11	0.75	0.17	0.38	0.08	0.24	0.24
Palpitations	2 (4.8%)	0 (0%)	0 (0%)	2 (25%)	0 (0%)							
Syncope	3 (7.1%)	1 (10%)	2 (14.3%)	0 (0%)	0 (0%)							
Dizziness	1 (2.4%)	0 (0%)	0 (0%)	1 (12.5%)	0 (0%)							
Dyspnoea	1 (2.4%)	0 (0%)	0 (0%)	0 (0%)	1 (10%)							
Dyspnoea + palpitations	1 (2.4%)	0 (0%)	0 (0%)	0 (0%)	1 (10%)							
ECG findings, *n*						*0*.*004*	0.49	*0*.*004*	0.17	***0***.***003***	0.33	*0*.*030*
Long QTc	1 (2.5%)	0 (0%)	0 (0%)	1 (12.5%)	0 (0%)							
Fragmented QRS	2 (5%)	0 (0%)	1 (7.1%)	1 (12.5%)	0 (0%)							
PVCs	4 (10%)	0 (0%)	0 (0%)	3 (37.5%)	1 (11.1%)							
RBBB	3 (7.5%)	0 (0%)	1 (7.1%)	0 (0%)	2 (22.2%)							
Negative T waves + long QTc	1 (2.5%)	0 (0%)	0 (0%)	1 (12.5%)	0 (0%)							
Negative T waves + PVCs	2 (5%)	0 (0%)	0 (0%)	2 (25%)	0 (0%)							
24 h Holter monitoring findings, *n*						0.074		0.15	0.73	***0***.***019***	0.42	0.097
PVCs > 5%	2 (8.33%)	0 (0.00%)	0 (0.00%)	1 (12.50%)	1 (12.50%)							
NSVT	6 (25.00%)	0 (0.00%)	0 (0.00%)	5 (62.50%)	1 (12.50%)							
VT	0 (0%)	0 (0%)	0 (0%)	0 (0%)	0 (0%)							
SCD, *n*	1 (2.4%)	0 (0%)	0 (0%)	1 (12.5%)	0 (0%)	0.23	—	0.25	—	0.18	—	0.25
Family history of SCD or VA, *n*	3 (7.5%)	2 (20%)	1 (7.1%)	0 (0%)	0 (0%)	0.31	0.35	0.21	0.16	0.47	0.41	—
Echocardiography												
LA volume (BP) index, mL/m^2^	32.1 (12.36)	26.1 (9.07)	29.3 (11)	28.7 (8.95)	46.3 (10.4)	*<0*.*001*	0.56	0.48	*0*.*002*	0.95	*0*.*003*	*0*.*003*
LV EDV (BP) index, mL/m^2^	53.7 (16.6)	50.2 (11.3)	48.3 (12)	57.7 (23)	61.4 (19)	0.24	0.99	0.85	0.51	0.67	0.28	0.98
LV ESV (BP) index, mL/m^2^	21.9 (9.7)	19.8 (8.3)	19.7 (6.7)	27.9 (15.9)	22.35 (7.1)	0.24	0.99	0.29	0.94	0.23	0.91	0.63
LV EF, %	62.4 (5.5)	64.5 (3.8)	61.9 (6.1)	60.5 (5.9)	62.4 (5.8)	0.49	0.29	0.11	0.33	0.58	0.86	0.48
Global longitudinal strain, %	20.6 (2.9)	21.9 (2.7)	19.8 (2.9)	19.3 (3.9)	20.9 (2.3)	0.29	0.077	0.46	0.92	0.92	0.22	0.25
Geometry parameters												
Annulus diameter, cm	3.47 (0.51)	3.05 (0.47)	3.46 (0.38)	3.86 (0.43)	3.59 (0.52)	*0*.*004*	*0*.*036*	*0*.*004*	*0*.*015*	0.060	0.41	0.26
Prolapsing leaflets, *n*						*<0*.*001*	*<0*.*001*	*<0*.*001*	<0.001	0.058	0.94	*0*.*047*
Anterior	5 (11.90%)	0 (0%)	3 (21.43%)	0 (0%)	2 (20.00%)							
Posterior	12 (28.57%)	0 (0%)	6 (42.86%)	1 (12.50%)	5 (50.00%)							
Bileaflet	15 (35.71%)	0 (0%)	5 (35.71%)	7 (87.50%)	3 (30.00%)							
Prolapse area (3Ch), cm^2^	0.47 (0.16–0.92)	0 (0)	0.49 (0.34–0.68)	1.29 (1.04–1.41)	0.49 (0.31–0.71)	*<0*.*001*	*<0*.*001*	*<0*.*001*	*<0*.*001*	***<0***.***001***	0.98	*0*.*003*
Prolapse height (3Ch), cm	0.44 (0.16–0.70)	0 (0)	0.47 (0.43–0.62)	0.84 (0.60–0.94)	0.42 (0.35–0.55)	*<0*.*001*	*<0*.*001*	*<0*.*001*	*<0*.*001*	***0***.***014***	0.12	*0*.*006*
MAD^c^, *n*	8 (19.05%)	0 (0%)	1 (7.14%)	6 (75.00%)	1 (10.00%)	*<0*.*001*	0.39	*<0*.*001*	0.30	***0***.***001***	0.80	*0*.*005*
Anterior leaflet length, cm	2.51 (0.51)	2.13 (0.23)	2.51 (0.53)	2.93 (0.55)	2.55 (0.41)	*0*.*008*	0.10	*<0*.*001*	*0*.*006*	0.14	0.79	0.25
Posterior leaflet length, cm	1.65 (0.56)	1.12 (0.23)	1.51 (0.50)	2.16 (0.38)	1.95 (0.49)	*<0*.*001*	*0*.*043*	*<0*.*001*	*<0*.*001*	***0***.***003***	0.084	0.31
Anterior leaflet thickness, cm	0.52 (0.43–0.65)	0.40 (0.37–0.5)	0.55 (0.47–0.65)	0.79 (0.65–0.85)	0.51 (0.47–0.61)	*0*.*002*	*0*.*008*	*0*.*001*	*0*.*049*	0.12	0.45	*0*.*010*
Posterior leaflet thickness, cm	0.50 (0.36–0.62)	0.33 (0.3–0.37)	0.45 (0.37–0.6)	0.72 (0.59–0.81)	0.59 (0.5–0.74)	*<0*.*001*	*0*.*006*	*<0*.*001*	*<0*.*001*	***0***.***003***	0.079	0.35
Traction parameters												
PM-MA distance ES, cm	2.93 (0.56)	2.66 (0.31)	2.72 (0.58)	3.46 (0.37)	3.03 (0.58)	*0*.*005*	0.64	*<0*.*001*	0.12	***0***.***005***	0.14	0.082
PM-MA distance PS, cm	2.46 (0.45)	2.51 (0.28)	2.46 (0.54)	2.22 (0.39)	2.61 (0.47)	0.32	0.26	0.12	0.82	0.45	0.37	0.099
PM-MA distance change, cm	−0.46 (0.45)	−0.15 (0.11)	−0.26 (0.13)	−1.25 (0.4)	−0.42 (0.18)	*<0*.*001*	0.63	*<0*.*001*	*0*.*04*	***<0***.***001***	*0*.*31*	*<0*.*001*
PM-MA distance change, %	14.73 (12.4)	5.6 (3.59)	9.46 (4.86)	36 (10.34)	13.7 (4.35)	*<0*.*001*	*0*.*042*	*<0*.*001*	*0*.*02*	***<0***.***001***	0.34	*<0*.*001*

Described as *n* (%), mean (SD), or median (IQR). Statistically significant *P* values were identified in italics. Statistically significant *P* values between the Benign MVP group and the Arrhythmogenic MVP group were identified in bold italicized values, which are the most remarkable findings of the study, mentioned in the text.

3Ch, three-chamber view; AMVP, arrhythmic MVP; BMI, body mass index; BMVP, benign MVP; EDV, end-diastolic volume; EF, ejection fraction; ECG, electrocardiogram; ES, early systole; ESV, end-systolic volume; LA, left atrium; LV, left ventricle; MA, mitral annulus; MAD, mitral annular disjunction; MR-MVP, MVP with severe mitral regurgitation; MVP, mitral valve prolapse; NSVT, non-sustained ventricular tachycardia; PM, papillary muscle; PS, peak systole; RBBB, right bundle branch block; SCD, sudden cardiac death; VA, ventricular arrhythmia; VT, ventricular tachycardia.

^a^MVP was defined as systolic displacement of one or both mitral leaflets ≥ 2 mm above the plane of the MA in the parasternal long-axis view.

^b^AMVP was defined as MVP combined with frequent or complex VA(≥5% total PVC burden, NSVT, VT, or VF) in the absence of any other well-defined arrhythmic substrate.

^c^MAD was defined as an abnormal longitudinal atrial displacement ≥ 2 mm of the hinge point of the mitral valve away from the ventricular myocardium during systole.

Regarding traction parameters, the AMVP group had the greatest PM-MA distance in early systole and the highest change in this distance between early and peak systole, compared to all the other groups.

These results support previously proposed AMVP characteristics, such as bileaflet involvement, large prolapse area and height, the presence of MAD, and pathological ECG and 24 h ECG Holter monitoring findings.^2,5^ Additionally, the findings in the MR-MVP group reinforce that significant MR is associated with symptoms, some degree of arrhythmic burden, and valve distortion.^11^

However, the most remarkable findings are those that differed between AMVP and BMVP. Specifically, the most interesting observation is that AMVP patients exhibited an increased PM-MA distance in early systole and a greater change of this distance between early and peak systole compared to BMVP (3.46 cm ± 0.37 vs. 2.72 cm ± 0.58, *P* < 0.01, and 36% ± 10.34 vs. 9.46% ± 4.86, *P* < 0.01, respectively). These findings were consistently maintained when comparing AMVP to controls and MR-MVP as well. In other words, patients with AMVP showed increased traction forces in the subvalvular apparatus compared to those with BMVP.

These results support the hypothesis that AMVP patients exhibit recognizable higher traction forces during systole, as reflected by the change in the PM-MA distance (*Figure 1*), and propose a possible novel intrinsic characteristic of this group. Although this mechanism was previously explored by Han *et al*.^12^ in a smaller cohort by magnetic resonance imaging, our results highlight the existence of excessive traction forces that can be quantified using an accessible and safe imaging method, such as TTE, and show parameters that could simply help in the distinction between BMVP and AMVP.

This study has certainly several limitations, mainly related to the number of patients and its retrospective nature. However, it prompts consideration of whether patients with increased traction forces should be the focus when assessing the risk of VA, although it remains uncertain if this represents a risk of potentially fatal malignant arrhythmias. While larger clinical trials are necessary to reach definitive conclusions, this preliminary proof-of-concept study demonstrates a significant correlation between augmented traction forces and VAs, aiding in the understanding of an infrequent and challenging condition.

## Data Availability

The data underlying this article will be shared on reasonable request to the corresponding author.
